# Hypertrophic Pachymeningitis in Chinese Patients: Presentation, Radiological Findings, and Clinical Course

**DOI:** 10.1155/2020/2926419

**Published:** 2020-08-14

**Authors:** Zhuajin Bi, Ke Shang, Jie Cao, Zhuyi Su, Bitao Bu, Shabei Xu, Chenchen Liu

**Affiliations:** ^1^Department of Neurology, Tongji Hospital, Tongji Medical College, Huazhong University of Science and Technology, Wuhan, Hubei, China; ^2^School of Public Health, Tongji Medical College, Huazhong University of Science and Technology, Wuhan, Hubei, China

## Abstract

**Background:**

Hypertrophic pachymeningitis (HP) is generally regarded as a rare inflammatory disease, which results in a diffuse thickening of the dura mater. We retrospectively collected data from patients with HP.

**Methods:**

A total of 16 patients with HP were included in our study. The clinical features, laboratory evaluation, imaging findings, treatment, and outcome were reviewed.

**Results:**

Of the 16 cases, half were male, with a mean age of 52.6 ± 13.2 years. The mean duration from onset to diagnosis was 8.6 months. The most frequent presenting symptoms in HP cases were a recurrently chronic headache (81.3%) and multiple cranial nerve injury (50%). Antineutrophil cytoplasmic antibody- (ANCA-) related HP was found in 5 cases and IgG4-related HP in 1 case. The intracranial pressure was elevated in 4 cases. The cerebrospinal fluid (CSF) had lymphocytosis in 5 cases and increased protein in 12 cases. Immunoglobulins (IgG, IgA, and IgM) and protein showed linear relationships in the CSF. On magnetic resonance imaging (MRI), localized or diffuse dura maters were thickened in all cases. HP combined with subacute subdural hemorrhage or hypertrophic spinal pachymeningitis was also observed in individual cases. Biopsy of the dura mater in one case showed amounts of inflammatory cells infiltrating, with an increased percentage of IgG4-positive plasma cells. Of all cases referring to glucocorticoid treatment, the symptoms have improved significantly in 10 cases. In other 6 cases, mycophenolate mofetil or azathioprine was added. All patients showed clinical improvement at the follow-up visits.

**Conclusion:**

The clinical characters of HP are chronic onset, recurrently chronic headache, and multiple cranial nerves paralysis. Inflammatory changes in CSF caused by intrathecal synthesis of immunoglobulin, characteristic dural enhancement on MRI, and pathologic biopsy are all helpful for diagnosis. The addition of immunosuppressant, especially mycophenolate mofetil, is a good choice for steroid-resistance HP.

## 1. Introduction

Hypertrophic pachymeningitis (HP) is a rare disorder characterized by localized or diffuse thickening of the dura mater and the inflammatory process of fibrosis, with clinical manifestations including chronic headache, multicranial nerve palsy, ataxia, and spinal cord dysfunction [1]. Existing knowledge from small case series shows HP can be considered to be an autoimmune disorder [2]. And abnormal immune indexes, including ANCA and IgG4 related antibody, often appear in patients with HP [3]. However, the mechanisms underlying HP are still undefined. In this study, we retrospectively collected data from patients with HP and explored their clinical features, laboratory test results, imaging findings, pathological examination, and disease outcome.

## 2. Methods

A total of 16 patients who were diagnosed with HP and admitted to our hospital from January 2013 to August 2019 were enrolled in our study. The diagnostic criteria for HP are defined as thickening and enhancement of the dura meter on T1-weighted enhanced MRI or dural biopsy. We excluded cases associated with intracranial hypotension and malignancy. Clinical, laboratory, neuroimaging, pathological, and therapeutic data of all cases were retrospectively collected.

Laboratory findings included erythrocyte sedimentation rate (ESR), c-reactive protein (CRP), T-SPOT.TB test, infectious and neoplasm markers, antinuclear antibodies (ANA), antidouble stranded DNA antibody (anti-dsDNA), rheumatoid factor (RF), and antineutrophil cytoplasmic antibodies (ANCA), which included perinuclear ANCA (p-ANCA), cytoplasmic ANCA (c-ANCA), and myeloperoxidase ANCA (MPO-ANCA). In addition, 7 cases were performed for serum IgG4 level. The cerebrospinal fluid (CSF) examination included intracranial pressure, cell count, protein, glucose, immunoglobulins (IgA, IgG, and IgM), bacterial and fungal stain, acid-fast staining, ink staining, CSF cultures, and exfoliation cytology. Brain magnetic resonance imaging (MRI) included T1, T2, fluid-attenuated inversion recovery (Flair), diffusion, and T1 with gadolinium enhancement. One case underwent a stereotactic surgical biopsy of the thickened dura.

SPSS 22.0 (Chicago, IL, USA) software was used for statistical analysis. Numerical data are presented as mean ± SD, and categorical data are presented as frequencies with absolute numbers and percentages. Relationships between immunoglobulins (IgA, IgG, and IgM) and protein in CSF were examined with Pearson correlation coefficients. A value of *p* < 0.05 was deemed to indicate statistical significance.

## 3. Results

### 3.1. Clinical Features

The 16 cases (8 males and 8 females) enrolled in the study were all inpatients at our hospital diagnosed with HP. They were between 24 and 68 years (the mean age was 52.6 ± 13.2 years). The mean duration from onset to diagnosis was 8.5 months, ranging from 1 month to 3 years. Of the 16 cases, 4 cases had a history of sinusitis, 4 cases complained of mastoiditis, 3 cases had undergone sinusitis or otitis media surgery, and 1 case admitted to traumatic brain injury.

The initial symptoms were headache in 13 cases, cranial nerve palsy in 8 cases, ataxia in 2 cases, and limb numbness in 2 cases. Of 8 cases with cranial nerve palsy, visual impairment due to optic neuropathy was the most frequent symptom, occurring in 5 cases. 4 cases had facial paralysis due to facial nerve injury. 3 cases had ophthalmoparesis due to III, IV, VI cranial nerve involvement, Auditory nerve and trigeminal nerve were also each affected in 3 cases (Table 1).

### 3.2. Laboratory Findings

On blood examinations, the WBC numbers increased in 4 cases, 10 cases showed an increased level of CRP (range 2.7 to 132.7 mg/L), and 8 cases had higher levels of ESR (range 18 to 111 mm/h). T-SPOT.TB tests were negative in all cases. 5 cases showed abnormalities in the autoantibody tests, including positive p-ANCA in 5 cases, positive MPO-ANCA in 4 cases, positive ANA in 3 cases, elevated levels of RF in 2 cases, positive anti-Ro-52 in 1 case, and elevated level of complement C4 in 1 case. All cases had normal results for specific infections and cancer screening (Table 1).

**Figure 1:**
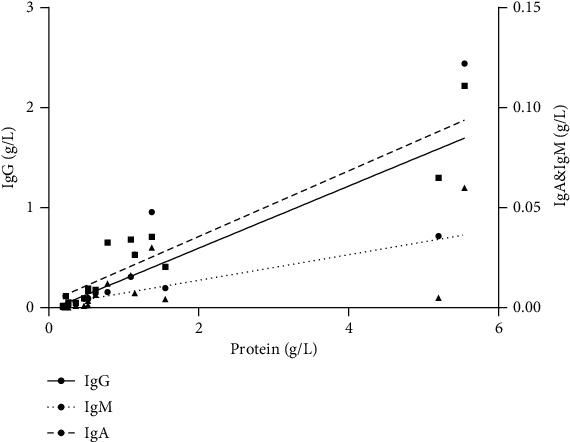
Scatter plot show a linear correlation between immunoglobulin (IgG, IgA, and IgM) and protein in CSF.

Lumbar puncture was performed in all cases. The intracranial pressure was elevated in 4 cases (range 200 to 290 mmH_2_O). There are 11 cases with abnormalities in CSF tests, including 5 cases with increased WBC count and 11 cases with an increased level of CSF protein. For CSF immunoglobulins, IgG was increased in 11 cases, IgA was elevated in 10 cases, and IgM was increased in 4 cases (Table S1 in the Supplementary Material). There were linear correlations between all immunoglobulins (IgG, IgA, and IgM) and protein in CSF (Figure 1: IgG = 0.311∗protein − 0.026, *p* < 0.01; IgA = 0.016∗protein + 0.002, *p* < 0.01; IgM = 0.006∗protein + 0.001, *p* < 0.01). CSF staining, cultures, and exfoliation cytology were all negative.

### 3.3. Neuroradiology and Histopathology

**Figure 2:**
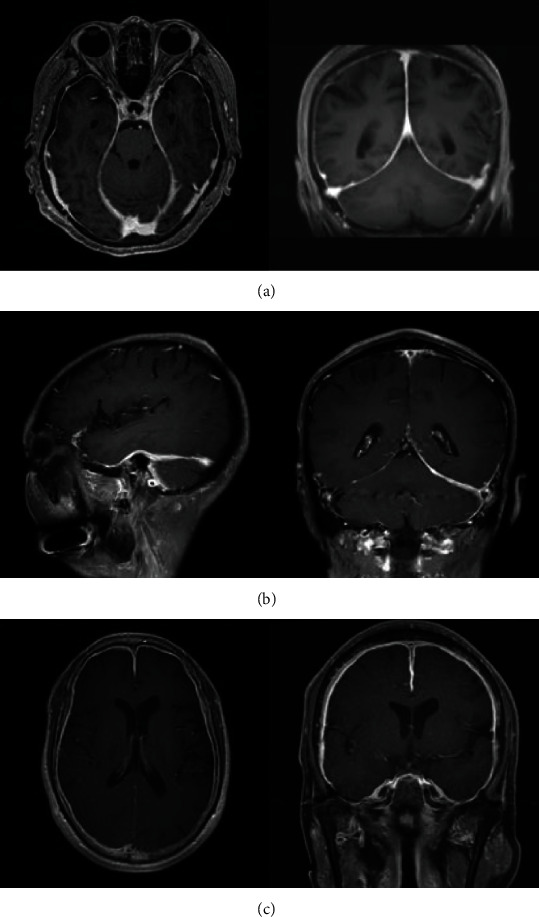
Neuroimaging of hypertrophic pachymeningitis. Case 4. Axial and coronal T1-contrasted MRI reveal enhancement of thickened falx cerebrum and tentorium cerebellum, mimicking the logo of Mercedes Benz (“Benz” sign) in coronal MRI (a). Case 5. Sagittal and coronal T1-contrasted MRI show thickening of left tentorium cerebellum, left frontal, and temporal dura mater (b). Case 6. Axial and coronal T1-contrasted MRI show diffuse linear dural enhancement, including the falx cerebrum (c).

**Figure 3:**
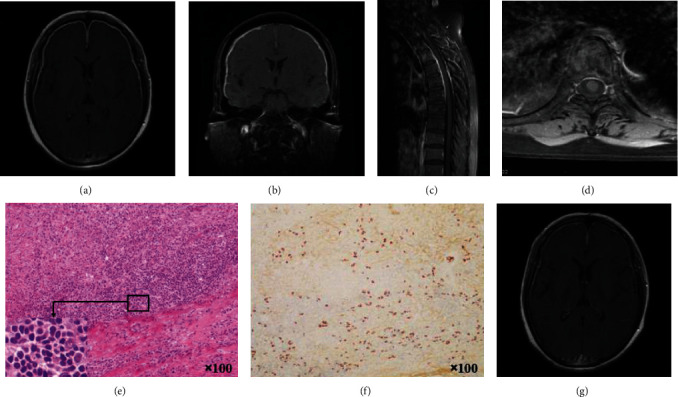
IgG4-related hypertrophic pachymeningitis involving the spinal dura mater (Case 8). Diffuse linear dural enhancement appears on axial (a) and coronal (b) T1-contrasted MRI. Sagittal (c) and axial (d) T1-contrasted MRI show dural thickening of the upper thoracic spinal dura mater. Pathologic slide of biopsy shows dense fibrosis and inflammatory cell infiltration (H&E 100×, (e)), with an increased percentage of IgG4-positive plasma cells (IgG4-IHC 100×, (f)). The dural enhancement is markedly reduced after steroid and immunosuppressive therapy (mycophenolate mofetil) on 4 mouths follow-up (g).

**Figure 4:**
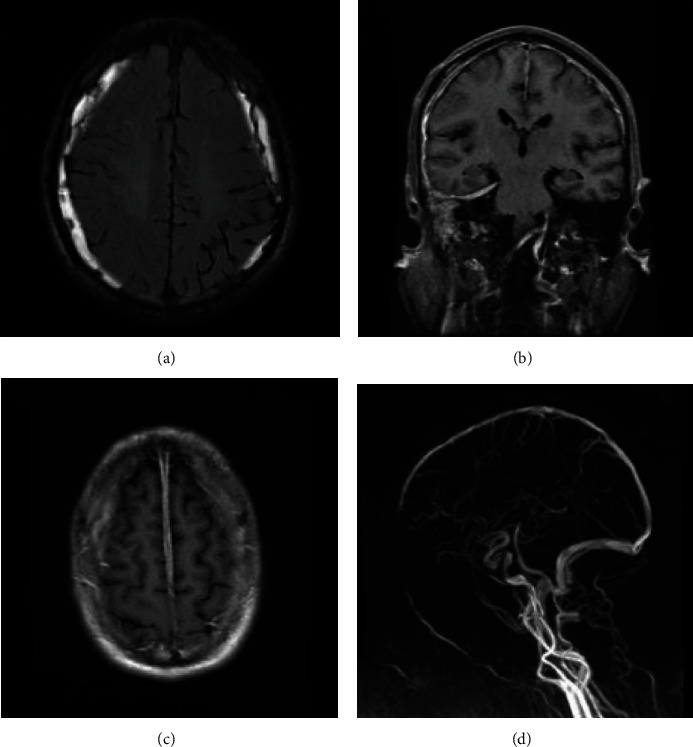
Hypertrophic pachymeningitis with subdural hemorrhage (Case 9). Bilateral subdural hemorrhage appears on axial T2-weighted fluid-attenuated inversion recovery imaging (a). Coronal T1-contrasted MRI reveals diffusion enhancement of the dura and right mastoiditis (b) and axial T1-contrasted MRI shows enhancement of the peripheral margin of the dura without central enhancement in frontal dura mater (c). Right otomastoiditis appears on MRI (b) and magnetic resonance venography was unremarkable (d).

Thickened dura was demonstrated by contrasted MRI in all cases. MRI results showed the diffuse dural enhancement in 5 cases, and local thickening with linear enhancement in tentorium or falx cerebri. The thickened dura was most prominent over the posterior part of the falx cerebri and the tentorium cerebelli, with “Benz” sign (Figure 2(a)). Coronal T1-contrasted MRI showed asymmetrical dural enhancement (Figure 2(b)) or symmetrical diffuse thickened dura (Figure 2(c)). HP can be combined with other lesions, such as hypertrophic spinal pachymeningitis (HSP) in case 8 (Figure 3) and subdural hemorrhage in case 9 (Figure 4). Magnetic resonance venography (MRV) was performed in 4 cases with intracranial hypertension and showed no dural arteriovenous fistula or dural sinus stenosis/occlusion. In addition, paranasal sinus MRI showed sinusitis and/or otitis media in 7 cases.

A stereotactic operation and biopsy of the hypertrophic dura mater was performed in case 8. Dural biopsy revealed fibrous tissue hyperplasia, chronic inflammation changes, diffuse lymphocytes, and plasma cells infiltration, without granulomas or vasculitis (Figure 3(e)). In addition, immunohistochemistry showed histological features with the infiltration of IgG4-positive plasma cells (Figure 3(f)).

### 3.4. Treatment and Prognosis

All cases were treated with corticosteroids. Among them, 9 cases received a pulse of high-dose methylprednisolone (0.5 g or 1 g), 4 cases were treated with methylprednisolone (40-80 mg/d), and other 3 cases received dexamethasone (10 mg/d). High-dose intravenous immunoglobulin was given to case 2 due to weakness. Clinical symptoms appeared to stabilize with no further deterioration in follow-up in 10 cases, while other 6 cases were added with immunosuppressive agents, including mycophenolate mofetil (case 5, 8, and 10) and azathioprine (case 6, 7, and 16). Among these, immunosuppressants were added in 3 cases due to adverse effects or poor efficacy of methylprednisolone during hospitalization. The other 3 cases had recurrent symptoms with steroids reduction or stopping after discharge. These symptoms improved on rehospitalization with the pulse of methylprednisolone and the addition of immunosuppressant. For laboratory tests, in cases 7 and 9, the CRP and ESR levels decreased to normal and testing for p-ANCA and MPO-ANCA transformed from positive to negative after corticosteroids treatment. Follow-up CSF tests were performed in 6 cases. The CSF protein returned to normal in 2 cases and decreased in 4 cases. MRI was reexamined in 10 cases during the 6-months follow-up, and the dural enhancements in neuroimaging were all markedly reduced.

## 4. Discussion

### 4.1. Etiology and Pathogenesis

HP is generally regarded as a rare inflammatory disease which results in a diffuse thickening of the dura mater. As we known, HP can be idiopathic or secondary to various other diseases. Several causes of secondary HP have been recognized, including infectious diseases, trauma, neoplasms, as well as autoimmune disorders [4–7]. The diagnosis of idiopathic HP depends on the exclusion of these possible etiologies for meningeal thickening. In this study, all cases had negative result in infections, sarcoidosis, and neoplasms screening. Although 5 cases showed abnormalities in the autoantibody tests, these cases lacked any other evidence for definite diagnosis of an autoimmune disorder. In summary, we tended to classify all subjects as idiopathic HP. Moreover, more than half of patients had a history of sinusitis or mastoiditis, even undergoing sinusitis or otitis media surgery. The role of otitis media or sinusitis in these patients is still uncertain. Shinichi et al. reported that the inflammation of otitis media or sinusitis might spread to the dura mater through direct invasion, labyrinthine route, or local circulation [8, 9]. However, we found dural lesions could occur in the contralateral side of otitis media or sinusitis. Also, the results of the microorganism culture in the CSF were all negative. Therefore, we speculated that the most possible mechanism may be related to the exaggerated immune response or immune disorder caused by these diseases, and further research on this topic is encouraged.

### 4.2. Clinical Characteristics

Previous studies showed HP predominantly affected men and occurred more common in 40-60 years old [10], also can be seen in children [6]. However, there is no male preponderance in our study. Headache was the most common symptom, and more than 80% of patients had headache in this study. HP patients always present with persistent swelling pain or paroxysmal tingling headache as the primary or only complaint, which may be due to chronic inflammatory stimulation or increased intracranial pressure caused by hypertrophic dura mater, cerebral edema, and blockage of the arachnoid granulations [11, 12]. Besides, cranial nerve palsy was another common symptom in HP, particularly of the optic nerve, auditory nerve, and abductor nerve in this study, which were associated with involvement of the dura surrounding the intracranial optic nerve and the cavernous sinus [11]. Kupersmith et al. reported that neurologic deficits were caused by fibrous entrapment of the nerve or localized ischemic damage due to nerve compression [13]. In our study, increased intracranial pressure, ataxia, and cognitive decline also appeared. This is consistent with the findings of other reports [11]. The clinical presentations appeared to be related with the location of the abnormal dura observed on gadolinium MRI. HP can present with epilepsy, ataxia, or dementia when partial obviously dural hypertrophy compressed brain parenchyma [14]. HP can affect the spinal dura mater as well as the cranial dura mater. Hypertrophic spinal pachymeningitis (HSP) usually have a predilection for the cervical and upper thoracic regions of the spine [15]. It is unusual for involving both the cranial and spinal dura mater simultaneously in HP [16]. Previous cases showed almost all patients with HSP presented with progressive paraparesis. In case 8, despite extensive involvement of the spinal dura mater (T1-T6), the symptom only showed numbness of both lower limbs. It was considered to be in the early stages of the disease, and no significant compression was observed on spinal MRI.

### 4.3. Laboratory Examination

HP association with the autoimmune disorders has been previously described. Among these disorders, ANCA and IgG4 are the most frequently recognized [3]. Iguchi et al. found several patients with HP were positive for ANCA and proposed the concept of ANCA-associated HP [4]. They also suggested that ANCA-positive HP could be considered as an early onset of ANCA-associated vasculitis in nervous system. Meanwhile, Chan et al. reported a case of IgG4-related sclerosing pachymeningitis which may be attributed to the IgG4-related disease spectrum [17]. Interestingly, in our study, 5 cases were positive for ANCA, these cases have elevated CRP and ESR levels, positive RF and ANA, and autoantibody testing transformed from positive to negative after treatment with glucocorticoids. All of these suggest HP as an autoimmune pathologic entity. However, all cases lacked any other evidence for the diagnosis of ANCA-associated vasculitis. Therefore, further studies are essential for evaluating whether ANCA-associated HP is an independent disease or a special type of ANCA-associated vasculitis.

Lumbar puncture can help to diagnose HP and evaluate prognosis. Nearly 70% patients have intracranial hypertension, increase in leukocyte numbers and protein levels in the CSF. All these findings confirm chronic inflammation in the CSF of HP. In addition, we found that the total amount of CSF protein was more than 5 g/L in two cases. In case 1, although such a large increase in CSF protein in HP is unusual, there was no evidence of leptomeningeal involvement, radiculopathy, or peripheral neuropathy, including tuberculosis and POEMS [18]. The level of CSF protein decreased from 5.2 g/L to 1.9 g/L after 1 week of corticosteroid treatment, and symptoms were also obviously relieved. In case 8, increased CSF protein could be explained by the extensive involvement of the upper thoracic spinal dura mater. Meanwhile, CSF immunoglobulins, including IgA, IgM, and IgG, were abnormal in varying degrees. Zhao et al. found that IgA and protein showed a linear relationship in CSF, but no linear correlation between IgM of IgG and protein [19]. They suggest dural inflammation involves the arachnoid and damage the blood-CSF barrier, which results in a proportion of immunoglobulins originate from the blood and then increase protein levels in the CSF. However, different from Zhao's research [20], we found linear correlations between all these immunoglobulins (IgG, IgA, and IgM) and protein in CSF. We believe intrathecal synthesis of immunoglobulin, comparing to protein, originates from the blood, may play a more important role in CSF inflammatory changes. An elevated IgG index in case 2 also confirmed s demonstrated the intrathecal synthesis of IgG. The mechanisms of the intrathecal synthesis of immunoglobulins are unknown [21]. Ikeda et al. reported that B cell activation factor of the tumor necrosis factor family and a proliferation-inducing ligand in the CSF may participate in the pathogenesis of HP through promoting autoreactive B cells [3]. Immunoglobulins may be secreted by activated immune memory cells.

### 4.4. Imaging and Pathological Features

Enhanced MRI is the most valuable test in the diagnosis of HP. The dura had diffused thickening that was most prominent over the posterior part of the falx cerebri and the tentorium cerebelli, with characteristic signs such as “Benz” sign or enhancement of the peripheral margin of the dura without central enhancement [10]. In general, linear dura enhancement suggests mild inflammation with abundant cell infiltration. Nodular enhancement represents the unequal dural hypertrophy, which may relate to the degree of repeated dural infection, and usually suggests poor prognosis [11, 19, 22]. No nodular enhancement in MRI was found in our study. It further explains the good prognosis in our cases with linear dura enhancement. Dura biopsy is the golden standard of HP confirmation. Histologic examination reveals that fibrous tissue hyperplasia and inflammatory infiltrates composed of lymphocytes and plasma cells. In addition, vasculitis, caseous necrosis, lymphoid follicle formation, or granulomatous changes have also been described in several cases [10, 11, 14, 16, 17]. Since the zone of most active inflammation always appeared along the periphery of the lesion, these histological presentations provide an explanation for the MRI characteristics. The low signal center represents a dense fibrosis, and peripheral enhancement represents active inflammatory reaction [10, 11]. What is more, in case 8, besides of the typical pathological manifestations of HP, we found an elevated levels of IgG4-positive plasma cells. This is consistent with the conclusion by Lindstrom et al. that increased numbers of IgG4-positive plasma cells in dura biopsy appeared in half of the patients with HP [14]. Chan et al. also proposes that a proportion of HP patients may be a part of the IgG4-related disease spectrum [17].

HP misdiagnosed as subdural hematoma is not uncommon, but HP presented as subdural hematoma is unusual [23]. In case 9, neuroimaging revealed subacute subdural hematoma combined with dural enhancement. It is necessary to differentiate HP and intracranial hypotension. Firstly, this case with persistent occipital distention pain, instead of orthostatic headache which usually caused by intracranial hypotension. What is more, this patient had a headache exceed 3 months, which is longer than the duration of subacute subdural hematoma. Then, multiple cranial nerve palsy increased CRP and ESR levels, positive ANCA, and abnormality in CSF, all of these were difficult to explain with the subacute subdural hematoma alone or low cranial pressure. At last, no active spinal CSF leak was found. The clinical and radiological presentation was improved after treatment of corticosteroids, instead of epidural blood patching or large dose solution infusion. Therefore, we speculate that inflammatory exudation of thickened dura may be the initial factor of the formation of subdural hematoma.

### 4.5. Treatment and Prognosis

To our knowledge, there is no consensus on the treatment for HP. Previous studies suggest that corticosteroid is the first choice for HP. The common method is started with a pulse therapy, then the dose of corticosteroid decreased gradually and eventually ceased all together after the symptoms have been relieved [24]. The immunosuppressive agents which can be chosen to prevent relapse have been reported in the literature, including azathioprine, methotrexate, cyclophosphamide, intraventricular cytarabine, and rituximab [6, 7, 22, 25]. However, there is a lack of reports on treatment with MMF. In the past decade, MMF has taken a central role in the treatment of several autoimmune disorders [26]. In our study, MMF is safe and effective for three patients with the condition who suffer from adverse effects or poor efficacy of corticosteroid. Therefore, we speculate that MMF can be effective and a therapeutic option in HP patients. We suggest that future research should focus on establishing a standard treatment strategy for this condition.

## 5. Conclusion

Here, we present clinical data from 16 patients with HP and our analyses. To conclude, chronic headache accompanied with multiple unexplained cranial nerve paralysis are the main presentations in HP. Intrathecal synthesis of immunoglobulin may play an important role in CSF inflammatory changes. The addition of immunosuppressant, especially mycophenolate mofetil and azathioprine, is a helpful treatment for steroid-resistance HP.

## Figures and Tables

**Table 1:** Clinical, laboratory, neuroradiologic, and therapeutic data from 16 patients with HP.

Case	Age/sex	Duration (months)	Previous history	Symptom	Laboratory test	CSF test		Lesions on MRI	Treatment	Follow-up
						WBC	Protein (g/mL)			
1	34/M	2	Sinusitis	Headache, CN VII	ESR↑	3	5200	Falx cerebri, tentorium	MP	SR
2	64/M	3	Otitis media surgery	Headache	CRP↑	14	785	Frontal and temporal dura, tentorium	DXM	SR
3	63/M	4	—	CN II, IV, VI	CRP↑	0	263	Right temporal dura	MP	SR
4	53/F	3	—	Headache, cognitive decline	—	0	213	Diffuse dural thickening	MP	SR
5	61/F	28	Mastoiditis	Headache, ataxia, CN II, VII, VII, XII	ESR↑, CRP↑, WBC↑, C4↑, p-ANCA (+), ANA (+)	10	472	Diffuse dural thickening	MP, MMF	RR
6	64/F	7	Sinusitis surgery	Headache CN II, III, VI, VII	ESR↑, CRP↑, p-ANCA (+), MPO-ANCA (+), ANA (+), RF (+), anti-Ro-52 (+)	0	627	Falx cerebri, tentorium	MP, Aza	SR
7	68/M	24	—	Headache	ESR↑, CRP↑, p-ANCA (+), MPO-ANCA (+), ANA (+), RF (+)	5	524	Frontal and occipital dura	MP, Aza	RR
8	49/F	36	Mastoiditis, sinusitis	Headache, CN II, V, limb numbness	ESR↑, CRP↑, p-ANCA (+), MPO-ANCA (+)	96	5548	Frontal and apical dura, falx cerebri, T1–T6	MP, HGG, MMF	RR
9	67/M	3	Mastoiditis	Headache, CN VII, VII	ESR↑, CRP↑, p-ANCA (+), MPO-ANCA (+)	60	1150	Falx cerebri, tentorium	MP	SR
10	37/M	6	Sinusitis	Limb numbness	—	4	524	Diffuse dural thickening	MP, MMF	SR
11	44/F	4	—	Headache	WBC↑	0	187	Falx cerebri, tentorium	MP	SR
12	41/F	5	Sinusitis	Headache	ESR↑, CRP↑, WBC↑	0	230	Diffuse dural thickening	MP	SR
13	58/M	4	Sinusitis surgery	Headache	CRP↑	2	1094	Falx cerebri	DXM	SR
14	62/F	3	—	Headache, ataxia CN II, VII, VII, XII	—	2	1557	Diffuse dural thickening	MP	SR
15	53/M	5	Traumatic brain injury, mastoiditis	Headache, CN X	ESR↑, CRP↑	20	1375	Tentorium	DXM	SR
16	24/F	1	—	Headache	WBC↑	1	361	Falx cerebri, tentorium	MP, Aza	SR

ANA: antinuclear antibodies; AZA: azathioprine; CN: cranial nerve; CSF: cerebrospinal fluid; DXM: dexamethasone; ESR: erythrocyte sedimentation rate; HGG: human gamma globulin; CRP: C-reactive protein (mg/L); Ig: immunoglobulin (mg/L); MMF: mycophenolate mofetil; MP: methylprednisolone; MPO-ANCA: myeloperoxidase antineutrophil cytoplasmic antibodies; p-ANCA: perinuclear antineutrophil cytoplasmic antibodies; RF: rheumatoid factor; RR: relapsing-remitting; SR: sustained remission; WBC: white blood cell.

## Data Availability

The data to support this study are available at the correspondence author upon request.

## Abbreviations

ANA: Antinuclear antibodies
ANCA: Antineutrophil cytoplasmic antibodies
CSF: Cerebrospinal fluid
CRP: C-reactive protein
ESR: Erythrocyte sedimentation rate
HP: Hypertrophic pachymeningitis
MRI: Magnetic resonance imaging
MMF: Mycophenolate mofetil
MPO-ANCA: Myeloperoxidase ANCA
p-ANCA: Perinuclear ANCA
RF: Rheumatoid factor
WBC: White blood cell.

## Supplementary Materials

Supplementary materials . Table S1: CSF findings in 16 cases with HP.
