# Supplementary material for: Hypertrophic Pachymeningitis in Chinese Patients: Presentation, Radiological Findings, and Clinical Course
Source: Biomed Res Int. 2020 Aug 14;2020:2926419. doi: 10.1155/2020/2926419 (PMC7448121; doi:10.1155/2020/2926419)
Supplement: Supplementary materials — . Table S1: CSF findings in 16 cases with HP. [file 2926419.f1.pdf]

Table S1: CSF findings in 16 cases with HP.

Case	ICP	WBC	Pandy	Pro	Glu	Cl	IgA	IgG	IgM
1	110	3	+	5200	8.13	116.7	64.9	716.0	4.9
2	135	14	-	785	2.85	124.1	32.6	157	12.1
3	120	0	-	263	4.13	122.2	2.7	21.8	0.2
4	115	0	-	213	3.72	127.0	0.9	15.2	0.2
5	130	10	-	472	4.79	130.4	4.7	88.9	0.9
6	150	0	+	627	3.77	129.5	8.0	143.0	6.5
7	140	4	+	524	4.58	119.6	8.4	96.1	3.5
8	280	96	+	5548	5.21	118.5	111.0	2440	60
9	265	60	+	1150	3.22	123.5	26.5	530	7.3
10	135	5	+	524	3.59	125.2	9.6	86.9	1.3
11	120	0	-	187	4.66	127.7	0.9	14.5	0.5
12	290	0	-	230	3.55	123.8	5.8	24.8	0.4
13	100	2	+	1094	4.27	128.0	34.1	309	16.2
14	260	2	-	1557	5.59	125.4	20.5	195	4.3
15	130	20	+	1375	3.93	126.2	35.5	955	30.1
16	120	1	-	361	3.23	126.1	2.5	32.6	1.0

Cl=chloride (mmol/L), CSF=cerebrospinal fluid, Glu=glucose (mmol/L), HP=hypertrophic pachymeningitis, ICP=intracranial pressure (mmH_2O), Ig=Immunoglobulin (mg/L), Pandy=Pandy's test, Pro=protein (mg/L), WBC=white blood cell ($\times 10^6$/L), +=positive, -=negative.
